# Microstructural Design and Processing Control of Advanced Ceramics

**DOI:** 10.3390/ma16030905

**Published:** 2023-01-17

**Authors:** Yu Chen, Qingyuan Wang

**Affiliations:** 1 School of Mechanical Engineering, Chengdu University, Chengdu 610106, China; 2 College of Architecture and Environment, Sichuan University, Chengdu 610065, China

Advanced ceramics are referred to in various parts of the world as technical ceramics, high-tech ceramics, and high-performance ceramics. They represent an important technology that has considerable impacts for a large variety of industries, branches and markets. It is considered as an enabling technology that has the potential to deliver high-value contributions for solving the challenges of our future. From a general point of view, the advanced ceramics sector comprises the following categories [1]:

(1) Functional ceramics: Electrical and magnetic ceramics (i.e., dielectrics, piezoelectrics, ferromagnetics), ionic conductors and superconductive ceramics.
(2) Structural ceramics: Monoliths and composites, e.g., oxides, nitrides, carbides, borides, and composite materials based on these materials.
(3) Bioceramics: e.g., hydroxyapatite and alumina.
(4) Ceramic coatings: Oxides, nitrides, carbides, borides, cermets and diamond-like coatings, deposited by technologies such as spraying, vapor deposition and sol-gel coating.
(5) Special glasses: Processed flat glass, fire resistant glazing and glasses for optoelectronics.

Figure 1 shows some representative advanced ceramics developed in recent years.

Advanced ceramics are usually composed of dense and fine-grained microstructures, thus can also be called fine ceramics. Due to their special mechanical performances and/or unique functional properties, advanced ceramics are useful in various applications covering thermal conductors, cutting tools, auto-motive/atomic energy/electronic/biomedical devices, energy conversion/sensor/actuation systems, and environmental and aerospace engineering. Japan Fine Ceramics Association (JFCA) published the “FC Roadmap 2050 (2021 edition)” in December 2021 [2]. The world market of the production of the fine ceramics industry is about USD 70 billion in 2018. Although the global production of automobiles and smartphones fell far short of the previous year’s level due to the impact of COVID-19 in recent years, fine ceramics output increased due to higher demand for semiconductors used in equipment, such as PCs and memory devices [3]. It is well known that after the outbreak of COVID-19 in 2020, ceramic materials have played an important role in combating the epidemic, especially piezoelectric ceramics, which play an important role in respirators, masks and advanced ultrasonic medical equipment (Figure 2). Besides the applications in the medicine field, piezoelectric ceramics can be also used as sensitive materials for various kinds of electroacoustic and piezoelectric devices, including sensors, detonators, micro-displacement actuators, ultrasonic transducers etc. In 2021, Global Industry Analysts, Inc published the latest global sales market forecasts of advanced ceramics. In the global sales market, China and the United States have large shares. An average annual growth of 6.3% is expected from 2020 to 2027 [4].

Advanced ceramics possess the tunable compositions and designable microstructures. First, advanced ceramics tend to lack a glassy component; i.e., they are “basically crystalline”. Second, microstructures are usually highly engineered, meaning that grain sizes, grain shapes, porosity, and phase distributions (for instance, the arrangements of second phases such as whiskers and fibers) can be carefully planned and controlled. Such planning and control require “detailed regulation” of composition and processing. Finally, such advanced ceramics with both well-designed microstructures tend to exhibit unique or superior functional attributes that can be “precisely specified” by careful processing and quality control [5]. There are many examples regarding the unique electrical properties such as excellent piezoelectricity, superconductivity or superior mechanical performances, including enhanced toughness or high-temperature strength, which were achieved by the microstructural design for ferroelectric/piezoelectric ceramics, bioceramics, structural ceramics, metal–ceramic composites, etc. [6,7,8,9,10,11,12].

The preparation of advanced ceramic components involves the heating process of ceramic powders, which must undergo special handling to control the heterogeneity, chemical compositions, purity, particle size, particle size distribution (PSD) and specific shape [13]. The aforementioned factors play a significant role in the properties of finished ceramic components. Moreover, the preparation of advanced ceramics usually involves more sophisticated processing steps. In short, processes become rather complex and can differ for various applications during the development of advanced ceramics [14]. The fabrication methods and processing conditions of advanced ceramics also impel their characteristics including excellent thermal properties, optical and electrical properties, corrosion-resistant, mechanical strength, hardness and anti-aging [15,16,17].

For example, in the editors’ laboratory, the PMN-29PT-1.6Gd ferroelectric ceramics were fabricated by a A-site modified oxide precursor method with two processing steps [18]. The removal of polar defect pairs with Gd-doping was considered to promote greater field-induced PNR reorientation and thereby increase the permittivity greatly. The combination of exquisite microstructural design and optimum processing control helped the ceramics to achieve an ultra-high piezoelectric coefficient (*d*_33_) up to 1210 pC/N, associated with a high dielectric permittivity of *ε*_r_ = 7059 at room temperature. Figure 3 shows the synthetic strategy of the PMN-29PT-1.6Gd ceramics designed by the authors.

Because of the attention to microstructural design and processing control, advanced ceramics are often high value-added products. Developments in advanced ceramic processing continue at a rapid pace, constituting what can be considered a revolution in the kind of materials and properties obtained.

This special issue contains ten papers that reported the results of several studies on functional ceramics, structural ceramics, ceramic composites, ceramic coatings and special glasses. The exquisite chemical compositions, novel and controllable fabrication process, subtle and designable meso/micro/nano-structures, unique physical and chemical properties as well as potential applications of these materials will be presented to the readers. 

The editors’ research group focused on the preparation, microstructures and electrical behaviors of the modified bismuth layer ferroelectric (BLSFs) ceramics. The authors of [19] studied the microstructures and electrical conduction behaviors of Gd/Cr co-doped Bi_3_TiNbO_9_ Aurivillius-phase ceramics. The authors of [20] reported the effects of oxide additives on the phase structures and electrical properties of SrBi_4_Ti_4_O_15_ high-temperature piezoelectric ceramics. The authors of [21] revealed the structures, electrical conduction and dielectric relaxation behaviors of the Gd/Mn co-doped CaBi_4_Ti_4_O_15_ Aurivillius-phase ceramics. By means of the chemical (substituting ions and oxide additives) modification and microstructural (phase composition and grain size/orientation) design for these three kinds of BLSFs ceramics, the electrical properties of materials were greatly improved. Such BLSFs ceramics are expected to obtain wide applications in the piezoelectric sensors with an operating temperature exceeding 500 °C.

The authors of [22] reported the microstructure and mechanical properties of composites obtained by spark plasma sintering of Ti_3_SiC_2_-15 vol.%Cu mixtures, and the microstructure and tribological properties of spark-plasma sintered Ti_3_SiC_2_-Pb-Ag composites at elevated temperatures were further investigated in [23]. The authors of [24] explored the effect of Al_2_TiO_5_ content and sintering temperature on the microstructure and residual stress of Al_2_O_3_-Al_2_TiO_5_ ceramic composites, and the effects of material design and sintering process on residual stresses were expounded from macro- and micro-levels. The authors of [25] studied the electron escape condition in semiconductor nanomaterials via photodeposition reaction. The authors of [26] prepared the boron nitride ceramic fibers containing amounts of silicon nitride using hybrid precursors of PBN and PCS via melt-spinning, curing, decarburization under NH_3_ to 1000 °C and pyrolysis up to 1600 °C under N_2_. The authors of [27], via a facile sol-gel method, explored the effects of the relative humidity (RH) on the BiFeO_3_ film in terms of capacitance, impedance and current-voltage (I–V). The authors of [28] investigated the tribological behaviors in Zr-based bulk metallic glass with a high heterogeneous microstructure.

In the manufacturing process of advanced ceramics, such as electronic ones, which have a high global market share, complicated physical and chemical changes occur. The characteristics of advanced ceramics, such as performance, reliability, and durability, are determined by the microstructure resulting from the transformation of the manufacturing process.

The experimental research and theoretic analysis aiming at some common scientific and technological problems in advanced ceramics presented above constitute the thematic scope of this Special Issue, entitled “Microstructural Design and Processing Control of Advanced Ceramics”. In conclusion, the published papers demonstrate the microstructures and processes’ relevance of the topics dealt with. The main purpose of this Special Issue is to publish significant papers presenting advanced research in the field of ceramic materials and ceramic composites with excellent functional and/or mechanical properties and broad applications.

## Figures and Tables

**Figure 1:**
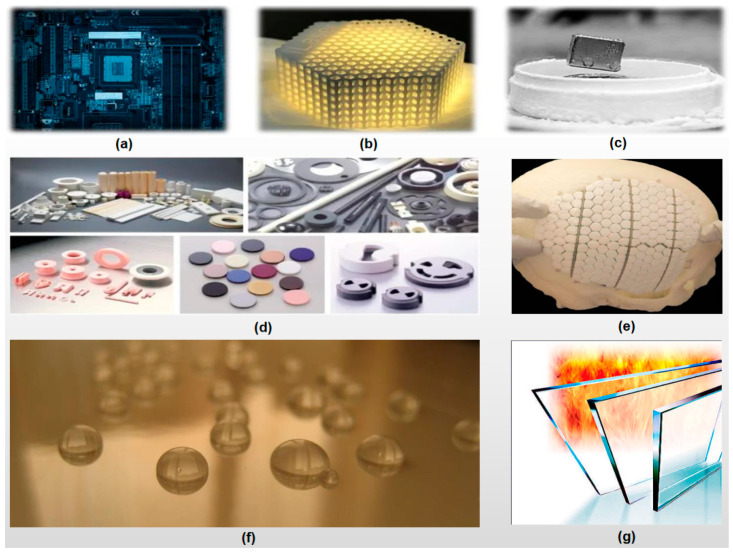
Some representative advanced ceramics developed in recent years: (**a**) Electronic ceramics, (**b**) 3-D printed high-strength ceramics, (**c**) Superconductive ceramics, (**d**) Various structural ceramics, (**e**) HAP bioceramics, (**f**) Nano-ceramic coatings, (**g**) Fire-resistant glasses.

**Figure 2:**
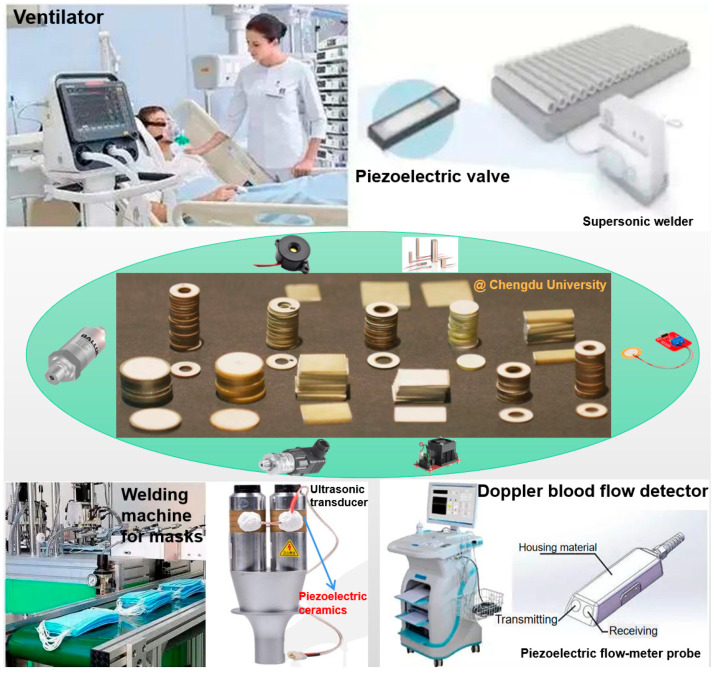
Applications of piezoelectric ceramics in the medicine field.

**Figure 3:**
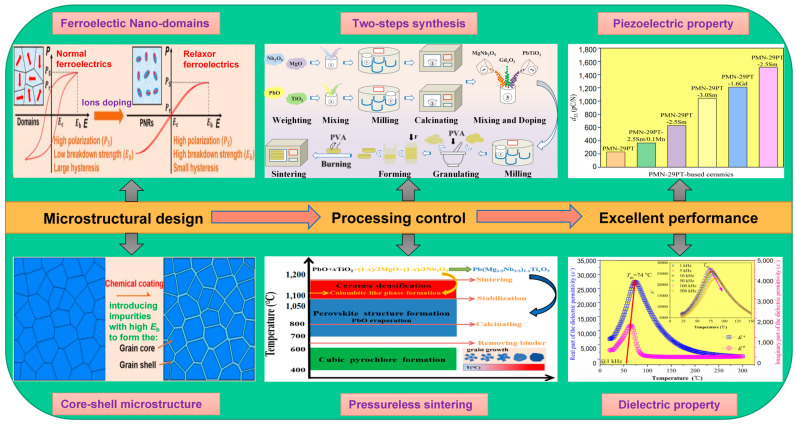
Synthetic strategy of the PMN-29PT-1.6Gd ferroelectric ceramics with excellent performances.
